# Antigen–antibody complex density and antibody-induced HLA protein unfolding influence Fc-mediated antibody effector function

**DOI:** 10.3389/fimmu.2024.1438285

**Published:** 2024-12-18

**Authors:** Tanusya Murali Murali, Yue Gu, Rabiatul Adawiyah Minhat, Jiawei Yap, Kathryn J. Wood, Cheng-I Wang, Nicholas R. J. Gascoigne, Vathsala Anantharaman, Paul Anthony MacAry

**Affiliations:** ^1^ Department of Microbiology and Immunology, Yong Loo Lin School of Medicine, National University of Singapore, Singapore, Singapore; ^2^ Immunology Translational Research Programme, Yong Loo Lin School of Medicine, National University of Singapore, Singapore, Singapore; ^3^ National University of Singapore-Cambridge Cell Phenotyping Centre, National University of Singapore, Singapore, Singapore; ^4^ Singapore Immunology Network, Agency of Science, Technology and Research, Singapore, Singapore; ^5^ Transplantation Research Immunology Group, University of Oxford, Oxford, United Kingdom; ^6^ Department of Medicine, Yong Loo Lin School of Medicine, National University of Singapore, Singapore, Singapore; ^7^ National University Centre for Organ Transplantation, National University Hospital, Singapore, Singapore

**Keywords:** alloantibodies, transplantation, antibody mediated rejection, human leukocyte antigen, antibody pathogenicity

## Abstract

Donor-specific antibodies (DSAs) targeting mismatched human leukocyte antigen (HLA) molecules are one of the principal threats to long-term graft survival in solid organ transplantation. However, many patients with long-term circulating DSAs do not manifest rejection responses, suggesting a degree of heterogeneity in their pathogenicity and related functional activity. Immunologic risk stratification of transplant recipients is complicated by challenges intrinsic to defining alloantibody responses that are potentially pathogenic versus those that are not. Thus, a comprehensive understanding of how human alloantibodies target and interact with donor HLA molecules is vital for the development and evaluation of new strategies aimed at reducing antibody-mediated rejection responses. In this study, we employ hydrogen–deuterium exchange–mass spectrometry (HDX–MS), molecular dynamics (MD) simulations, and advanced biochemical and biophysical methodologies to thoroughly characterize a panel of human monoclonal alloantibodies and define the influence of Fc-region biology, antibody binding kinetics, target antigen density, and structural characteristics on their ability to potentiate the forms of immune effector mechanisms that are strongly implicated in transplant rejection. Our findings have significant implications for our understanding of the key biological determinants that underlie the pathogenicity or lack thereof of human alloantibodies.

## 1 Introduction

Kidney transplantation (KTx) is the current optimal treatment for patients with end-stage renal disease (ESRD) due to an observed superiority of outcomes linked to quality of life and mortality in recipients (1). There remains a global shortage of organs available for transplantation, and thus, extending their survival and functionality is of the utmost importance. While acute forms of renal transplant rejection have decreased significantly in recent years due to the employment of successful immunosuppression (2), chronic rejection remains a significant obstacle to long-term graft survival. Alloimmune responses targeting mismatched human leukocyte antigens (HLAs) play an important role in initiating the graft injury responses linked to chronic rejection. In particular, the presence of donor-specific alloantibodies (DSAs) is correlated with a higher risk of graft rejection (3) and a reduction in long-term graft survival in KTx (4).

Alloantibodies can influence the rejection of transplanted organs through common Fc-mediated immune effector mechanisms including the recruitment of complement (5, 6) and the mobilization of FcR-bearing immune effector cells that mediate antibody-dependent cellular cytotoxicity (ADCC) (7, 8). It has also been intimated that dynamic interactions between DSAs and target HLA molecules can result in the activation of endothelial cells and thus cause direct injury to the allograft (9). However, DSAs have also been observed in many long-term transplant cohorts where they are tolerated. This indicates that not all alloantibody responses are equally harmful (10). Moreover, our current understanding of paratope–epitope relationship(s) in human alloantibody responses usually relies upon low-resolution approaches that analyze antibody binding patterns for HLA alleles in *polyclonal* human serum where confounding factors such as the hook effect, weak interactions with non-specific antibodies, or complement components can influence the quality of the analyses (11, 12). Previous studies have suggested that antibody-mediated immune responses are potentially influenced by the antibody isoform, its ability to form immune complexes (13), antigen density and availability of the target antigen, and/or the antibody binding kinetics (14, 15).

The current state-of-the-art platforms that are employed to detect donor-specific, anti-HLA alloantibodies rely upon solid-phase assays to measure antibody binding patterns for HLA alleles in polyclonal human serum combined with binding-site prediction algorithms that utilize HLA amino acid sequence alignment and/or stereochemical modeling (11, 12). The main limitation of this approach is that it only reveals the presence of DSAs but is weakly predictive of how these potentially influence graft injury/rejection in an individual patient. This translates into a low-resolution analysis that affects the risk stratification of transplant recipients and consequently results in the sub-optimal application of immunosuppression. Thus, there is a growing literature that supports a thorough *functional* characterization of alloantibodies to distinguish those that are pathogenic with the aim of improving our management of antibody-mediated rejection (ABMR) (6).

In this study, we provide a detailed structure/function analysis of a repertoire of human monoclonal anti-HLA alloantibodies. We show that monoclonal alloantibodies can be organized into two groups based on their strong versus weak immune-potentiating activity and compare the influence of allostery, antigen–antibody complex density, binding kinetics, and structural interactions in determining their relative potential pathogenicity. Our principal goal was to identify specific measurable parameters that can be utilized to better differentiate pathogenic versus non-pathogenic alloantibodies in the future.

## 2 Materials and methods

### 2.1 Phage-Fab panning

A naive phage-Fab library (Humanyx Pte. Ltd., Singapore) was used to pan against recombinant monomers as previously described (16). HLA monomers at a concentration of 20 μg/mL were coated in a MaxiSorp immunotube (Cat. #341866; Thermo Scientific, Waltham, MA, USA) at 4°C overnight. The tube was blocked with 4% skim milk (Cat. #1706404; Bio-Rad, Hercules, CA, USA) in phosphate-buffered saline (PBS). Pre-blocked Humanyx phage library was added to the coated immunotube and incubated for 1.5 hr at room temperature. Two rounds of negative selection with streptavidin were included in the panning process to remove any non-specific phages. Tubes were then washed multiple times with 4% milk in PBST (PBS in 0.05% Tween20), followed by four rounds of PBST and twice with PBS. Bound phages were eluted by trypsinization at 37°C for 0.5 hr. *Escherichia coli* TG1 was then infected with the eluted phages and cultured in 2YT broth containing 2% glucose. Once the OD_600nm_ reached 0.5, the TG1 cells were co-infected with M13K07 helper phage (Cat. #N0315S; NEB, Ipswich, MA, USA) for 0.5 hr at 37°C. The culture was then replaced with fresh 2YT (Cat. #CUS-4042-1kg; First Base, Axil Scientific, Singapore) containing 100 μg/mL of carbenicillin (Cat. #10177012; Gibco, Grand Island, NY, USA) and 25 μg/mL of kanamycin (Cat. #10177012; Gibco), followed by overnight incubation at 30°C and 200 rpm. The next day, phages were precipitated out using 20% PEG/2.5 M NaCl (Cat. #A17541.30; Thermo Fisher, Waltham, MA, USA). An aliquoted phage was used for subsequent panning.

### 2.2 Polyclonal phage ELISA

Monomers at a concentration of 4 μg/mL were coated onto an ELISA plate (Cat. #32296; SPL Life Sciences, Pochon, Korea) and incubated overnight at 4°C. The plate was washed twice with 1× PBS before being blocked with 380 μL/well of 4% skim milk (in 1× PBST) for 1.5 hr at room temperature. The plate was washed twice with PBST and further incubated with 100 μL of polyclonal phages collected from each pan (diluted 1:10 in 4% skim milk) for 1 hr at room temperature. The plate was washed four times with PBST, and 100 μL of anti-M13-HRP secondary antibody (Cat. #11973-MM05T-H; Sino Biological, Beijing, China) diluted 1:2,500 in 2% skim milk was added. The plate was incubated in the dark for 1 hr at room temperature. TMB (Cat. #34029; Thermo Scientific) was added, and the reaction was stopped after 10 min with 1 M sulfuric acid. Absorbance was measured at 450 nm.

### 2.3 Monoclonal phage ELISA

Approximately 600 single Fab phages were cultured individually in 96-well “U” bottom plates containing 2YT broth supplemented with 2% glucose and 100 μg/mL of carbenicillin until the OD_600nm_ reached 0.5. These cultures were then infected with M13K07 helper phage for 0.5 hr at 37°C. The plate was spun down, and the culture was then replaced with fresh 2YT containing 100 μg/mL of carbenicillin and 25 μg/mL of kanamycin, followed by overnight incubation at 30°C and 200 rpm for phage expression. The following day, the culture supernatant containing phage was tested for binding with the HLA monomer of interest on ELISA as described above. The plasmid of positive clones (OD_450nm_ above 0.5) was extracted with E.Z.N.A Plasmid DNA Mini kit (Cat. #D6942-02; Omega Bio-Tek, Doraville, GA, USA) and sent for sequencing.

### 2.4 Antibody expression and purification

Variable regions of the heavy chain (V_H_) and light chain (V_L_) were digested and ligated into their respective plasmids. Plasmids (ptt5) containing constant regions of either a human or mouse IgG1 were used for the expression of fully human or mouse chimeric monoclonal IgG1. Heavy- and light-chain plasmids were co-transfected into a HEK293T using branched polyethyleneimine (Cat. #408727; Sigma-Aldrich Corp., St. Louis, MO, USA). Antibodies were harvested 1 week later and purified using the Protein G Sepharose Fast Flow resin (Cat. #17061801; Cytiva Life Science, Marlborough, MA, USA).

### 2.5 Sodium dodecyl sulfate–polyacrylamide gel electrophoresis

Purified antibody at a mass of was diluted in 4× Laemmli Sample Buffer (Cat. #1610747; Bio-Rad) and incubated at 95°C for 10 min before being loaded into a 12% resolving gel. The gel was then stained with Coomassie Brilliant Blue and de-stained with water.

### 2.6 Binding of alloantibodies to cell surface HLAs using flow cytometry

Approximately 0.5 × 10^6^ of CF863-C*07:02 B-Lymphocyte cell line was incubated with 1 μg of antibody (and positive/negative control) for 1 hr. Anti-Class I HLA mouse antibody (W6/32) (Cat. #HB-95; ATCC, Manassas, VA, USA) and 14c10-IgG1 (anti-dengue antibody) were used as the positive and negative control, respectively, when specified. Cells were washed and further incubated with secondary antibody [Goat anti-Mouse IgG1 Cross-Adsorbed Secondary Antibody, Alexa Fluor™ 647, Cat. #A-21240 (Invitrogen, Carlsbad, CA, USA) or Goat anti-Human IgG1 Cross-Adsorbed Secondary Antibody, Alexa Fluor™ 647, Cat. #21445 (Invitrogen)] for 1 hr. Cells were washed and analyzed on an Attune NxT flow cytometer. The FlowJo software was used for data analysis.

### 2.7 Binding of antibodies on ELISA

Different Class I HLA monomers at a concentration of 4 μg/mL were coated onto a MaxiSorp plate for 2 hr at room temperature. The plate was washed with 1× PBS twice and blocked with 380 μL/well of 4% skim milk in 1× PBS for 1 hr at room temperature. The plate was washed thrice with 1× PBS/T before being incubated with 4 μg/mL of purified antibodies diluted in blocking buffer for 1 hr at room temperature. The plate was washed thrice with 1× PBS/T before the addition of a secondary antibody (Thermo Fisher Scientific) diluted 1:10,000 in blocking buffer and incubated for 1 hr, protected from light. TMB was added, and the reaction was stopped after 10 min with 1 M sulfuric acid. Absorbance was measured at 450 nm.

### 2.8 Complement-dependent cytotoxicity assay

Forty microliters (50k cells) was seeded per well in a 96-well U bottom plate and incubated with the antibody for 1 hr at 37°C. Human monoclonal anti-dengue IgG1 monoclonal antibodies were used as isotype control (17). Baby rabbit complement (Cedarlane, Cat. #CL3441-S) at a volume of 5 μL (final concentration 10%) was added to the plate and further incubated for 4 hr at 37°C. The plate was spun at 450 g for 5 min; 25 μL of supernatant was discarded, and cells were resuspended in 150 μL PBS for further washing. The plate was spun again at 450 g for 5 min, and 140 μL of supernatant was discarded. 7-Aminoactinomycin D (7-AAD) (Cat. #A1310; Invitrogen) was diluted in 1× PBS [containing 3% fetal bovine serum (FBS)] to a final concentration of 20 μg/mL. Cells were resuspended with 25 μL of 7-AAD to each well and incubated for 20 min at room temperature. The sample was topped up with 150 μL of FACS buffer before analysis on flow cytometry. The FlowJo software was used to analyze the data. The 7-AAD^high^ population was identified as dead cell target cells. Percentage cytotoxicity was calculated as follows: (experimental cell death − baseline cell death)/(maximum cell death − baseline cell death) × 100%). A two-tailed Mann–Whitney test was used to compare the experimental antibodies to the isotype control.

### 2.9 Antibody-dependent cell-mediated cytotoxicity assay

NK cells were isolated from peripheral blood mononuclear cells (PBMCs) using STEMCELL Easysep Human NK Cell Isolation Kit (Cat. #19665; STEMCELL Technologies Inc., Vancouver, BC, Canada). PBMCs were resuspended in 1× PBS (containing 1% FBS) at 5 × 10^7^ cells/mL and transferred to a sterile FACS tube. Isolation was performed according to the manufacturer’s instructions. NK MACS medium (Cat. #130-114-429; Miltenyi Biotec, Bergisch Gladbach, Germany) was used for the expansion of NK cells for 7 days. Target cells CF863-C*07:02 were prepared by centrifuging at 450 g for 5 min and resuspending at 1 × 10^6^ cells/mL in sterile 1× PBS. CellTrace CFSE cell proliferation dye (Cat. #C34570; Invitrogen) was added to cells (1:1,000 dilution) and incubated at 37°C for 20 min, with gentle agitation every 5 min. An equal volume of warm FBS was added to quench the reaction after 20 min and further incubated for 5 min at 37°C. Cells were pelleted by centrifuging at 450 g for 3 min and resuspended with Roswell Park Memorial Institute (RPMI) media (Cat. #11875119; Gibco) to a final concentration of 1 × 10^6^ cells/mL. Twenty microliters (20k cells) was seeded per well and incubated with antibodies for 1 hr at 37°C. Anti-dengue IgG1 monoclonal antibodies were used as isotype control. NK cells were added at 80k per well (1:4 target cell:effector cell ratio). The assay was incubated for 4 hr at 37°C. Cells were pelleted by centrifuging at 450 g for 3 min. 7-AAD was diluted in 1× PBS (containing 3% FBS) to a final concentration of 20 μg/mL. The supernatant was discarded, and cells were resuspended in 50 μL of diluted 7-AAD. The plate was incubated for 20 min at room temperature. The sample was topped up with 150 μL of FACS buffer before analysis on flow cytometry. The CFSE+7-AAD^high^ population was identified as dead target cells. Percentage cytotoxicity was calculated as (experimental cell death − baseline cell death)/(maximum cell death − baseline cell death) × 100%. A two-tailed Mann–Whitney test was used to compare the experimental antibodies to the isotype control.

### 2.10 Estimation of HLA density using QIFIKIT

Target cells CF863-C*07:02 were counted to prepare 0.25 × 10^6^ cells for each alloantibody (and positive/negative control). The Falcon tube was spun down at 300 g for 5 min, and pellets were resuspended in RPMI media. Approximately 5 μg of alloantibody (in 20 μL of 1× PBS) was incubated with 0.25 × 10^6^ cells at 4°C for 1 hr. Resuspended (vortex) beads at a volume of 100 μL from Vials 1 and 2 were placed in two separate tubes. Washing buffer (1% FBS in 1× PBS) at a volume of 3 mL was added to beads and cells. Tubes were centrifuged at 300 g for 5 min. The supernatant was discarded. Goat-anti mouse secondary antibody was added to all the tubes and incubated for another 45 min at 4°C. Cells and beads were washed with 1 mL washing buffer (1% FBS in 1× PBS), and the plate was spun down at 300 g for 5 min. The supernatant was discarded, and pellets were resuspended with buffer before analysis on flow cytometry.

### 2.11 IgG affinity measurement by Bio-layer interferometry

The binding affinity of purified antibodies to HLA-A*11:01, HLA-B*40:01, or HLA-C*07:02 antigen was measured on the Octet96Red system. The amine-reactive (AR2G) sensor tips (ForteBio, Menlo Park, CA, USA) were activated in freshly prepared 20 mM 1-ethyl-3-(3-dimethylaminopropyl)-carbodiimide hydrochloride (EDC) and 10 mM *N*-hydroxysuccinimide (NHS) solution, and each antibody was immobilized to the sensor tips using a concentration of 5 μg/mL in 10 mM sodium acetate pH 6 buffer. When immobilization was completed, the sensors were quenched in 1 M ethanolamine pH 8.5, followed by establishing a stable baseline in kinetics buffer [phosphate-buffered saline buffer supplemented with 0.1% Tween-20 and 0.1% bovine serum albumin (BSA)]. Association and dissociation were then measured by dipping the IgG-immobilized sensor tips into different concentrations of their respective antigen from 1 μM to 15.625 nM (*200 nM to 12.5 nM for B40+streptavidin complex*) in twofold dilutions for 6 min, followed by kinetics buffer for 10 min. Assays were run at 30°C, and data were analyzed on the Octet System Data Acquisition Software version 9.0.0.4 using the 1:1 fitting model.

### 2.12 Hydrogen–deuterium exchange mass spectrometry

All HLA and antibody complexes were formed by incubating 600 μg HLA with antibodies 1A6, 1H2, 2H7, and 2G11 individually in a ratio of 1:1. Deuterium exchange buffer was prepared by drying aqueous PBS buffer pH 7.4 to remove all H_2_O, and 99.9% D_2_O was added to reconstitute to form the deuterium exchange buffer. Amide hydrogen–deuterium exchange reaction was initiated by adding apo HLA, apo antibody, or HLA–antibody complexes into a 10× volume of deuterium exchange buffer to give a final D_2_O concentration of 89.9%. Deuterium labeling was then carried out at four different deuterium exposure times: 1 min, 10 min, 30 min, and 100 min. Hydrogen–deuterium exchange (HDX) reaction was quenched by reducing the pH of the reaction to 2.5 by adding NaOH in GnHCl and tris(2-carboxyethyl)phosphine-hydrochloride (TCEP-HCl) to achieve a final concentration of 1.5 M GnHCl and 0.25 M TCEP-HCl. Quenching of the reaction was performed for 1 min on ice to further slow down any back-exchange reaction. All deuterium exchange reactions were performed in triplicates, and the final deuteron uptake value for each peptic digested fragment reported is an average value of the triplicates without back-exchange correction. All quenched samples were injected into the nano-UPLC HDX sample manager (Waters, Milford, MA, USA), as previously described (Wales et al., 2008). Pepsin digestion was performed using a Waters Enzymate BEH pepsin (2.1 × 30 mm) column in 0.05% formic acid in water at 100 mL/min. Proteolyzed pepsin fragment peptides were trapped by a 2.1 × 5 mm C18 trap (ACQUITY BEH C18 VanGuard Pre-column, 1.7 mm, Waters, Milford, MA, USA) and eluted with an 8%–40% gradient of acetonitrile in 0.1% formic acid at 40 mL/min into a reverse-phase column (ACQUITY UPLC BEH C18 Column, 1.0 × 100 mm, 1.7 mm, Waters) by nanoACQUITY Binary Solvent Manager (Waters, Milford, MA). Peptides were ionized by electrospray into SYNAPT G2-Si mass spectrometer (Waters, Milford, MA) acquired in MS^E^ mode for detection and mass measurements. [Glu1]-fibrinopeptide B ([Glu1]-Fib) at a concentration of 200 fmol/mL was simultaneously injected into the mass spectrometer at a flow rate of 10 mL/min for continuous calibration during sample acquisition. Peptides of HLA and all antibodies were identified using mass spectra of undeuterated samples and searched against a database containing the respective amino acid sequence of the proteins separately using the PROTEIN LYNX GLOBAL SERVER version 3.0 (Waters, Milford, MA) software with the settings of precursor ion mass tolerance of <10 ppm, and products per amino acids of at least 0.1 with a minimum intensity of 500 for both precursor and product ions were selected. Three undeuterated replicates per protein were collected, and the final peptide list was generated using peptides identified in at least two out of three replicates. All deuterium uptake calculations were performed using the DYNAMIX 3.0 software (Waters, Milford, MA). Deuteron uptake per peptide was obtained by subtracting the mass centroid of deuterium-exposed peptides (1 min, 10 min, 30 min, and 100 min) from the mass centroid of the undeuterated peptide. Differences between the HLA–antibody complex with their apostates were then obtained by subtracting deuteron uptake of all peptides in the HLA–antibody complex state with their respective apostates and represented as a difference plot of peptides spanning from N- to C-terminus. Differences between states were considered significant if deuteron uptake exceeded the threshold of greater or lesser than 0.5 deuterons.

## 3 Results

### 3.1 Fc-mediated immune functionality correlates with the density of HLA–antibody complexes on the target surface

**Figure 1:**
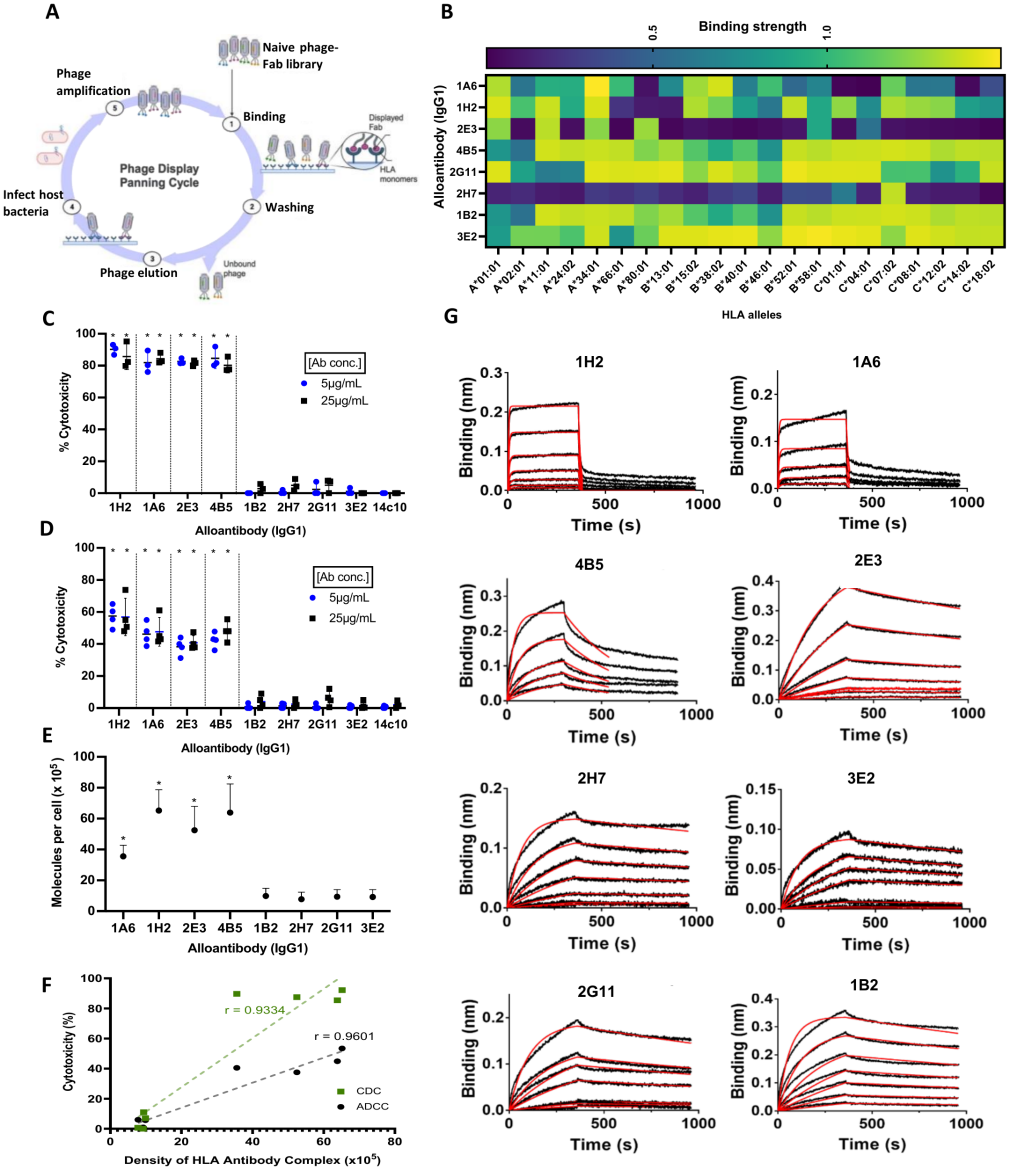
Characterization of immune-potentiating/pathogenic alloantibodies. **(A)** Bio-panning workflow for antibody discovery using the Humanyx phage library Created with BioRender.com. **(B)** Binding reactivity of the eight alloantibodies against 21 Class I alleles tested on ELISA. **(C)** Complement-dependent cytotoxicity (CDC) using CF863-C*07:02 as target cells. **(D)** Antibody-dependent cellular cytotoxicity (ADCC) using CF863-C*07:02 as target cells. Cells were incubated with complement serum for CDC or with NK cells (effectors cells) for 4 hr before analysis. Two different concentrations, 5 and 25 μg/mL, were tested for all the antibodies (means ± s.d., N= 4 independent experiments). *p = 0.0286 when analyzed with two-tailed Mann–Whitney test, compared to isotype control human monoclonal anti-dengue antibody 14c10-IgG1 for both CDC and ADCC. **(E)** Human leukocyte antigen (HLA)–antibody complex on CF863-C*07:02 cell line as measured using QIFIKIT. Results represent four independent experiments. *p = 0.0286, when analyzed with two-tailed Mann–Whitney test, compared to Ab 1B2-IgG1 (highest mean in the non-pathogenic group). **(F)** Strong correlation was observed between cytotoxicity and antigen–antibody complex density (Pearson’s correlation coefficient, r = 0.9334 for CDC and r = 0.9601 for ADCC). **(G)** Binding kinetics measured on Bio-layer interferometry (BLI) biosensor. Association and dissociation curves of the eight alloantibodies. Six different concentrations of HLA-A*11:01 and/or HLA-C*07:02 monomers, ranging from 1 μM to 15.6 nM, with two fold dilutions were tested. HLA-B*40:01–strep complex was tested at concentrations ranging from 200 nM to 12.5 nM. Red line shows 1:1 curve fitting for each concentration. Results represent two independent experiments.

A combinatorial human phage-Fab library was employed to isolate and clone recombinant human monoclonal alloantibodies (**Figure 1A**). Specifically, our phage-Fab library (Humanyx Pte. Ltd.) was panned against expressed and purified monomeric forms of Class I heterotrimers, specifically HLA-A*11:01, B*40:01, and HLA-C*07:02. Polyclonal ELISA results revealed significant enrichment in sequential panning prior to monoclonal alloantibody isolation and cloning (**Supplementary Figure 1A**). A total of eight alloantibodies (Abs) were selected and expressed as fully human monoclonal IgG1s expressing heavy- and light-chain polypeptides of 50 kDa and 25 kDa, respectively (**Supplementary Figure 1B**). For comparison, we employed a defined alloantibody 2E3 that was previously described (18). The differential binding profiles of the recombinant alloantibodies against 21 different Class I HLA monomers were measured by ELISA. Five of the eight antibodies—1A6-IgG1, 1H2-IgG1, 4B5-IgG1, 1B2-IgG1, and 3E2-IgG1—displayed broad cross-reactivity by binding to 17 of the 21 forms of HLA tested. Antigenic determinants that are shared between HLA types have been widely reported based on the high degree of sequence identity (greater than 90%) between HLA alleles. Thus, cross-reactivity in the binding profiles of alloantibodies is expected. Three of the tested alloantibody clones exhibited restricted reactivity based on binding patterns to fewer HLA alleles (**Figure 1B**).

To investigate the Fc-mediated immune functionality of each monoclonal alloantibody, we performed complement-dependent cytotoxicity (CDC) and ADCC assays on tissue-typed human target cells displaying the relevant HLA molecules—these are two key mechanisms that have been described in alloantibody-mediated pathogenicity and transplant rejection (19). **Figure 1C** illustrates the level of cytotoxicity (expressed as percentile killing) caused by the tested alloantibodies. CF863-C*07:02, an Epstein–Barr virus (EBV)-derived human B-lymphocyte cell line, was used as the target for these assays. This cell line expresses HLA-A*11:01, HLA-B*51:02, HLA-B*55:01, HLA-C*07:02, and C*14:02. CDC results reveal that four of the tested Abs can recruit complement-mediated killing. Abs 1A6-IgG1, 1H2-IgG1, 2E3-IgG1, and 4B5-IgG1 induced between 80% and 90% complement-mediated cytotoxicity of the target cells. In contrast, Abs 2H7-IgG1, 1B2-IgG1, 2G11-IgG1, and 3E2-IgG1 exhibited weak or negligible complement-mediated killing. Similar observations were made when ADCC assays were conducted to measure NK cell-mediated killing of target cells. Abs 1A6-IgG1, 1H2-IgG1, 2E3-IgG1, and 4B5-IgG1 induced significant (38% to 54%) NK cell-mediated cytotoxicity (**Figure 1D**), while Abs 2H7-IgG1, 1B2-IgG1, 2G11-IgG1, and 3E2-IgG1 were not able to induce ADCC. These data suggest that immune-potentiating, pathogenic mechanisms involving Fc-mediated responses (such as CDC and ADCC) can differ significantly between antibodies targeting HLA molecules displayed on the same cellular targets.

We next investigated the potential role of antigen–antibody complex density in antibody pathogenicity, as it was consistently highlighted in the literature, especially in the context of therapeutic antibodies targeting tumor antigens (14, 15). We wanted to understand if the same could be observed in the characteristics of pathogenic alloantibodies. To thoroughly investigate the role of antigen density in influencing the functionality/pathogenicity of alloantibodies, we conducted a detailed quantitation of the concentration of HLA–alloantibody complexes bound to the surface of the target cell line, CF863-HLA-C*07:02. This involved employing principles of advanced protein engineering to convert the eight human alloantibodies into mouse IgG1 chimeras with similar binding profiles. **Figure 1E** shows the numbers of HLA–antibody molecules per target cell measured by QIFIKIT. The pathogenic antibodies displayed a higher level of complexes with a mean density per cell of 65,168, 63,836, 52,452, and 35,571 for Abs 1H2, 4B5, 2E3, and 1A6, respectively. In contrast, the non-pathogenic antibodies measured a mean of 9,872, 9,395, 9,212, and 7,782 for Abs 1B2, 2G11, 3E2, and 2H7, respectively. In summary, pathogenic alloantibodies exhibited four to 10 times higher numbers of antibody–HLA complexes per cell than that measured of the non-pathogenic antibodies. This translates into a clear correlation between antigen–antibody complex density on the target cell surface and complement/NK cell-mediated cytotoxicity (**Figure 1F**). It is important to note that while the total cell-surface antigen density (based on numbers of HLA molecules) on the target cells utilized for these experiments remain equivalent, the number of epitope sites available for binding by an individual alloantibody is based on both the specificity and binding capacity of the individual alloantibody. Hence, this read-out reveals a differential density of alloantibody–HLA complexes rather than differences in the numbers of individual HLA molecules. Different binding capacities explain why some comparatively cross-reactive antibodies such as Abs 1B2 and 3E2 measured relatively low densities of HLA–antibody complexes despite being cross-reactive for most of the HLAs displayed by the target cell line.

### 3.2 Analyzing the influence of binding kinetics on alloantibody pathogenicity

To determine the role of alloantibody binding kinetics on pathogenicity, we conducted a detailed biophysical analysis of the eight alloantibodies by Bio-layer interferometry (BLI). As immobilizing antigen (followed by binding/association with IgGs) typically measures avidity, we reversed the orientation to immobilize IgGs, followed by measuring binding/association with antigen. This allows for a more accurate assessment of affinity for whole IgGs (20). Sensorgrams of the association and dissociation curves and the kinetic values for the eight alloantibodies on their target HLA molecules are shown in **Figure 1G**, **Supplementary Table 1**, respectively.

All eight alloantibodies measured a mean dissociation equilibrium constant (K_D_) of 10^−7^ to 10^−8^ molar (M), indicating a relatively high affinity. The group of non-pathogenic antibodies displayed values between 17.6 nM and 39.4 nM, displaying better affinities toward their respective HLAs compared to the three pathogenic antibodies, namely, Abs 4B5, 1H2, and 1A6, which measured 20.8 nM, 357 nM, and 643 nM, respectively.

The dissociation constant (k_off_) measures how fast the antibody dissociates from its antigen. The values measured for the non-pathogenic antibodies were in the range of 3.24–3.76 × 10^−4^ s^−1^. Antibody 2E3 from the pathogenic group also measured 3.70 × 10^−4^ s^−1^, similar to the non-pathogenic antibodies. However, three of the four pathogenic antibodies (Abs 1A6, 1H2, and 4B5) measured values between 6.86 × 10^−2^ s^−1^ and 3.55 × 10^−3^ s^−1^, suggesting faster dissociation compared to the others.

The non-pathogenic antibodies measured association constants (k_on_) between 9 × 10^3^ and 2 × 10^4^ M^−1^ s^−1^, despite taking a similar time to dissociate. Antibody 2E3, from the pathogenic group, once again followed the trend of the non-pathogenic antibodies. Interestingly, the remaining pathogenic antibodies measured values at ~1.31–2.49 × 10^5^ M^−1^ s^−1^, implying that these antibodies exhibit a faster rate of binding to their target HLAs.

Taken together, these data indicate that the non-pathogenic antibodies displayed higher affinities toward their respective HLAs compared to the pathogenic antibodies. This suggests that higher affinity binding of alloantibodies does not translate into increased pathogenicity. In addition, no significant difference was observed when comparing the k_on_ and k_off_ values of the two groups. While three of the four pathogenic antibodies, namely, Abs 1H2, 1A6, and 4B5, displayed a similar pattern of fast binding and dissociation, the other pathogenic antibody (namely, Ab 2E3) exhibited similar values to the non-pathogenic antibodies, 2G11 and 3E2. The absence of a correlation between the affinity/dissociation constant and immune potentiation suggests that antibody binding kinetics is not predictive of pathogenicity.

### 3.3 The binding of pathogenic antibodies promoted an overall increase in structural stability for the HLA–antibody complex

To investigate allostery and dynamic interactions that underlie alloantibody binding to HLA-C*07:02 molecules, we employed HDX–mass spectrometry (HDX–MS) at proximal and distal sites of HLA–alloantibody interaction. The principal advantage of this method is that it allows an analysis of a whole IgG molecule (as opposed to FAB), and the interactions take place in a soluble substrate. This represents a more accurate representation of the physiological conditions under which these interactions usually take place. We chose two alloantibodies from each group: namely, Abs 1A6-IgG1 and 1H2-IgG1 from the pathogenic group and Abs 2H7-IgG1 and 2G11-IgG1 from the non-pathogenic group. The structural changes in the HLA molecules as a result of alloantibody binding are measured by a decrease and/or increase in deuterium uptake—this reflects changes in solvent accessibility and allostery. Dynamic protein modeling can then be utilized to map these changes across the 3D structure of the HLA molecules.

Sixty-four peptides were identified overall, covering approximately 82% of the HLA sequence (**Supplementary Figure 2A**). Four time points—1 min, 10 min, 30 min, and 100 min—were probed to identify any conformational changes that occurred over time. Evaluation of the deuterated HLA-C*07:02 molecule in the absence of an antibody found the antigen-binding cleft to be highly dynamic, as shown by the Relative fractional (RFU) uptake value (**Supplementary Figure 2B**).

**Figure 2:**
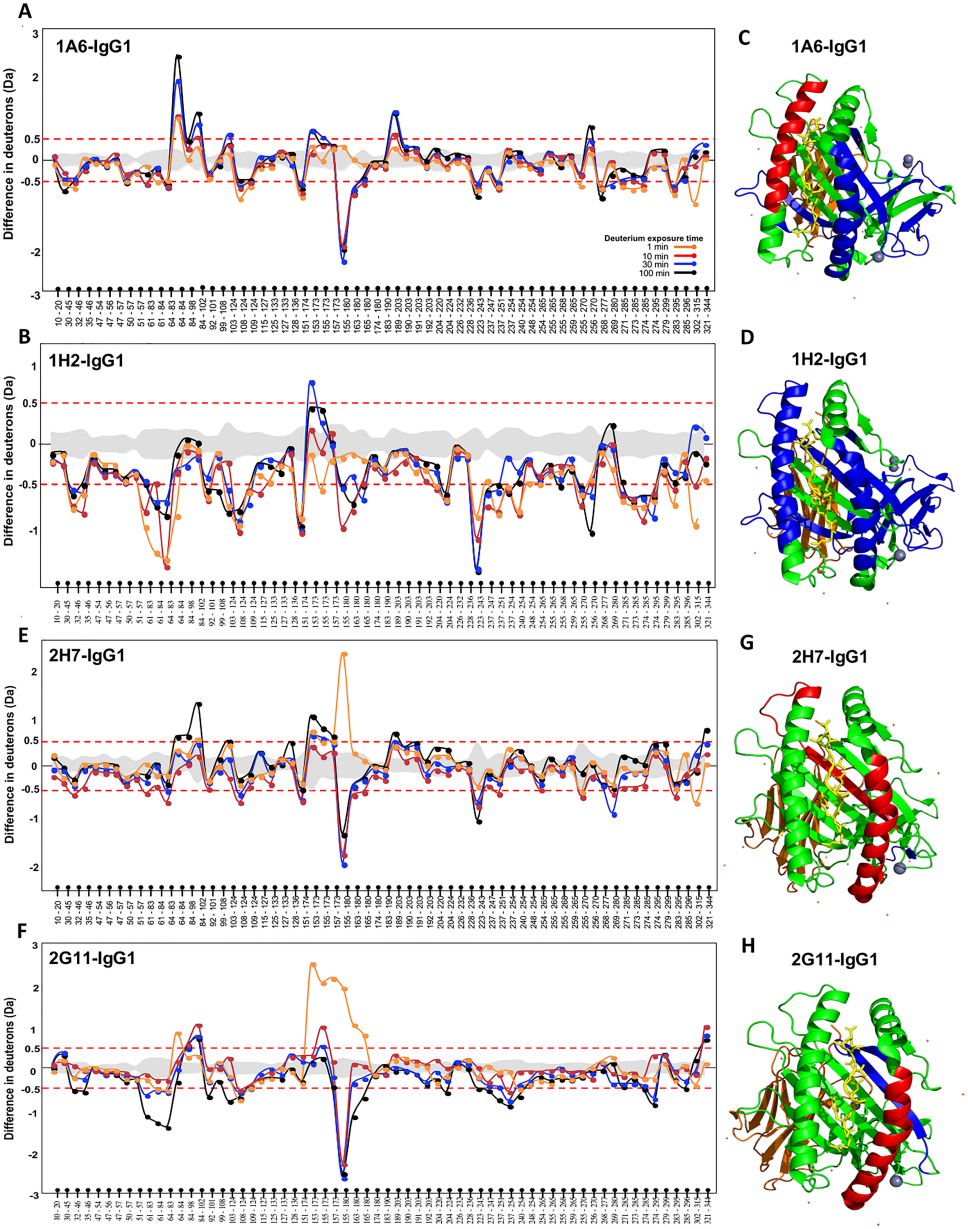
Hydrogen–deuterium exchange (HDX) profile of HLA-C*07:02. The difference in deuterium uptake of apo human leukocyte antigen (HLA) vs. HLA bound to Abs **(A)** 1A6-IgG1, **(B)** 1H2-IgG1, **(E)** 2H7-IgG1, and **(F)** 2G11-IgG1. The y-axis represents the difference in deuterons, while the x-axis represents the peptides identified, starting from the N- to C-terminal of the HLA. The gray-shaded area in between the red dotted lines represents the standard deviation derived from three experiments. The red dotted line represents the threshold above which differences in deuterium uptake between bound and unbound (free) HLA were considered significant. This threshold is reported to be approximately 0.5 Da for all peptides. Crystal structure of HLA-C*07:02 is highlighted in blue, and red represents reduction and increase in deuterium uptake at 1 min for Abs **(C)** 1A6-IgG1, **(D)** 1H2-IgG1, **(G)** 2H7-IgG1, and **(H)** 2G11-IgG1.

**Figures 2A, B** show the HDX profiles of pathogenic antibodies Ab 1A6-IgG1 and Ab 1H2-IgG1 binding to HLA-C*07:02, respectively. We analyzed the difference in deuterium uptake when bound by the pathogenic Abs. One important observation made was the decrease in deuterium uptake at peptide 155-180 located at the alpha (α) 2 region, suggesting stabilization in this region upon the binding of pathogenic antibodies. It should be noted that the α2 region forms part of the antigen-binding cleft. As binding occurs within the first minute, we analyzed the difference in deuterium uptake and mapped it on the HLA-C*07:02 structure as represented in **Figures 2C, D**. In addition, we also evaluated the overall HDX profile of HLA across the four time points when bound to the pathogenic antibodies. We found that the binding of Abs 1A6 and 1H2 resulted in a decrease in deuterium uptake, as can be observed from the 1-min time point across the different regions of the HLA molecule. Most of these regions remain contracted throughout the four time points, suggesting structural stability of the HLA–Ab complex from the first minute. However, the difference in terms of deuteron decrease was more profound in Ab 1H2 compared to Ab 1A6. It can be seen that stabilization of the complex is achieved upon the binding of pathogenic antibodies.

The HDX profile of the non-pathogenic antibodies Ab 2H7-IgG1 and Ab 2G11-IgG1 binding to HLA-C*07:02 is shown in **Figures 2E, F**, respectively. Interestingly, changes were once again observed at peptide 155-180. At 1 min, there was a significant deuterium uptake of more than two deuterons, upon the binding of both the non-pathogenic antibodies. However, the subsequent time points revealed a significant decrease in deuterium uptake. This suggests that the binding of Ab 2H7 triggers unfolding in that region as observed at the 1-min time point. Soon after, the region becomes more contracted as observed at 10 min onward. In terms of the overall HDX profile, most of the deuterium decrease only occurred at a later time point, at 10 min for Ab 2H7, which eventually reverted back to its original state toward the 100-min time point. In contrast, for Ab 2G11, significant changes could only be observed at the 30-min and 100-min time points and in much lesser regions compared to the pathogenic Abs. This is probably because the changes are so subtle that their cumulative effect can only be observed at a much later time point. **Figures 2G, H** show the HLA structure when bound to the non-pathogenic antibodies at the first minute, which once again reiterates the instability of the Ab–HLA complex. As such, the overall structural stability of the HLA–Ab complex can be ranked as follows: from the most to least stable, 1H2 > 1A6 > 2G11 > 2H7, which directly correlates with pathogenicity.

## 4 Discussion

In this study, we describe the isolation and functional characterization of eight monoclonal human alloantibodies derived from a human phage-Fab library, which was panned against three Class I HLA monomers, namely, HLA-A*11:01, HLA-B*40:01, and HLA-C*07:02. We then grouped these into pathogenic and non-pathogenic antibodies (four antibodies in each group) based on their immune-potentiating activity. In brief, pathogenicity was predicated on the ability of the antibodies to cause CDC and/or ADCC, two of the most important mechanisms of antibody-mediated rejection in transplantation. One notable observation was that antibodies that induced CDC were also able to cause ADCC, suggesting that these Fc-mediated immune responses may be influenced by the same biological determinants. We proceeded to investigate three of the principal factors that are believed to dictate the pathogenicity of antibodies based on previous studies (14, 21–23). These factors are defined as antigen density, binding kinetics, and biophysical interactions. To our knowledge, this is the first such study to do a direct comparison between alloantibodies for a thorough evaluation of the influence of these different factors in influencing pathogenicity.

First, we measured antigen–antibody complex density using the QIFIKIT, which utilizes the quantitative flow cytometry method and is commonly used for measuring antigen density per cell using flow cytometry. However, it is important to note that the number of molecules measured in this experiment is based on the ability of each alloantibody to form a complex with HLA on the cell surface. We quantified the amount of HLA–Ab complex on the surface of the CF863-HLA-C*07:02, the same cell line that was used for the cytotoxicity assays. We found that there was a significant difference in the number of complexes that were measured between the two groups. The pathogenic antibodies measured four to 10 times higher than those measured of the non-pathogenic alloantibodies, exhibiting a clear correlation between the density of the HLA–Ab complex on the cell surface and antibody pathogenicity. It was also noted that Ab 1H2, which recorded the highest cytotoxicity in both CDC and ADCC, also had the highest antigen–antibody complex density compared to the other antibodies. We hypothesize that high complex density may have provided more binding sites, thereby allowing the formation of antibody hexamers, which have been shown to be crucial in the activation of the complement cascade (24–26). Similarly, polyvalent immune complexes of antigen–antibody are required for the assembly and activation of Fcγ for ADCC (13), which is only possible with high antigen–antibody complex density.

Next, we measured the binding kinetics of the eight alloantibodies using the BLI biosensor to understand its correlation to pathogenicity. This label-free method allows real-time kinetics measurements of interactions between nucleic acids, peptides, proteins, small molecules, and lipids (27). Our results revealed that all non-pathogenic antibodies had better affinities compared to the pathogenic antibodies, indicating little correlation between affinity and pathogenicity. Analysis of the association and dissociation constant values further revealed no conclusive evidence that could distinguish the two groups. While several past studies reported the relevance of slow-off rates and its correlation to pathogenicity (15, 28), we, contrastingly, observed fast on- and off-rates for at least three of the four pathogenic antibodies that we tested, indicating that pathogenicity may not be reliant on the antibody kinetics alone.

**Figure 3:**
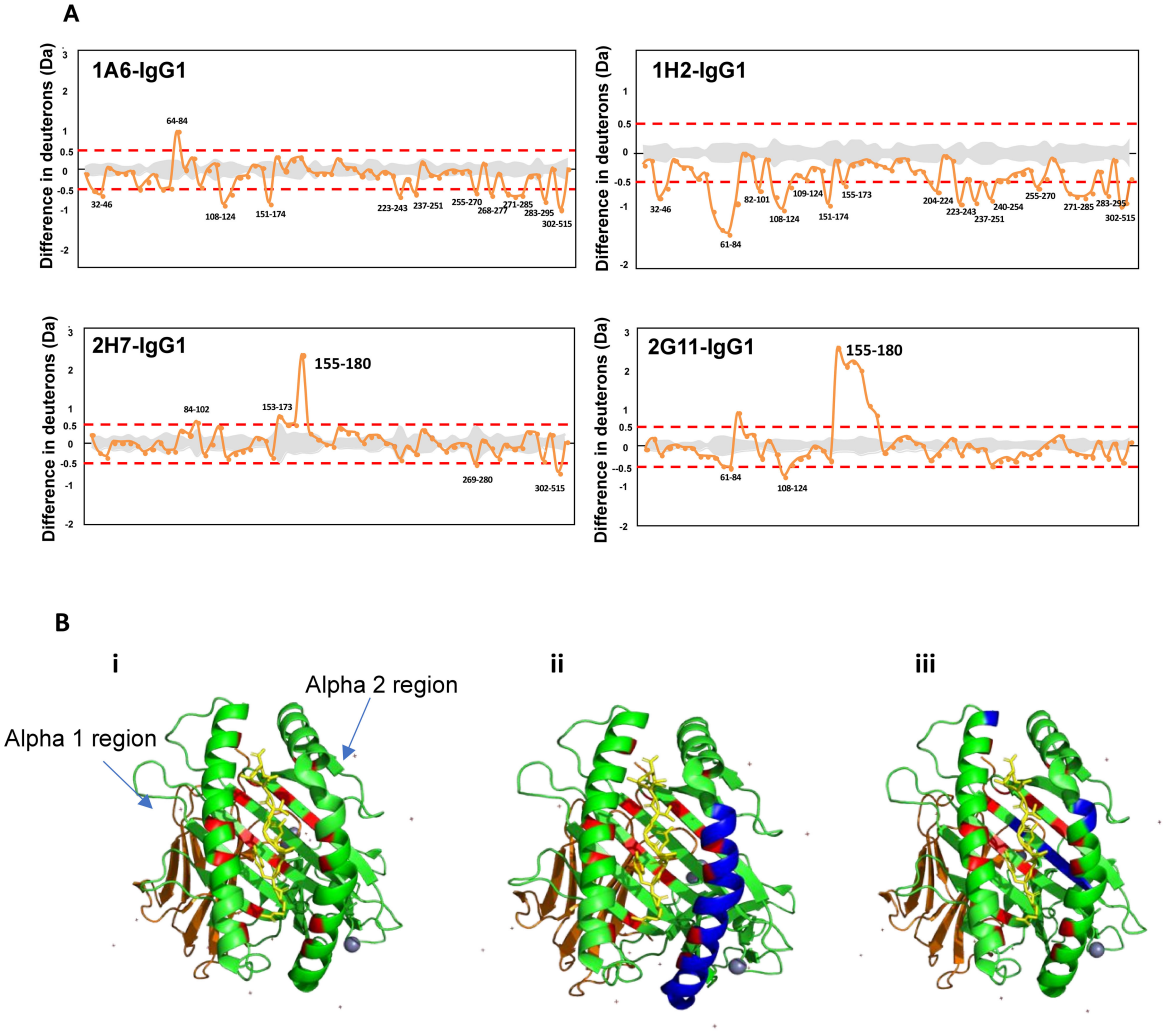
Structural comparison of uptake in deuterium when bound to the non-pathogenic vs. pathogenic antibodies. **(A)** Hydrogen–deuterium exchange (HDX) profile of alloantibodies binding to human leukocyte antigen (HLA) at the first minute. **(B)** Crystal structure of HLA-C*07:02 (PDB ID: 5VGE). The green ribbons represent the alpha (α) 1, 2, and 3 regions, with the peptide sticks highlighted in yellow and β2m in orange. The regions highlighted in red are parts of the antigen-binding cleft that interact with the peptide. Regions highlighted in blue represent the uptake of deuterium in (i) unbound HLA-C*07:02, (ii) HLA when bound by non-pathogenic antibodies, and (iii) HLA when bound by pathogenic antibodies.

Finally, we investigated the biophysical properties of the HLA–antibody interaction using the HDX–MS method. We analyzed any structural changes that occurred to the HLA molecules and compared the two groups to distinguish the interaction of pathogenic and non-pathogenic Abs. The most significant observation made was the uptake of deuterium, at the α2 region during the first minute when bound by the non-pathogenic Abs (**Figure 3A**). In MHC Class I, peptides are bound between the α1 and 2 regions (antigen-binding cleft) by forming hydrogen bonds with the surrounding amino acid residues within the HLA molecule (29). A study by Kaur et al. reported the anchoring of the peptide for HLA-C*07:02 located at residues P1-Arg, P2-Tyr, P3-Tyr, and P9-Leu (30). To understand how the unfolding at region 155-180 affects the antigen-binding cleft, we mapped the interacting amino acid residues (in red) onto an HLA-C*07:02 structure, as illustrated in **Figure 3B**. The unfolding (highlighted in blue) occurred at a region where at least four amino acids are interacting with the peptide. As such, we hypothesize that the unfolding at the antigen-binding cleft may have loosened the grip on the peptide, thereby affecting the stability of the HLA–Ab complex. Past studies have reported that HLA-C*07 is among the unstable HLA-C variants in a peptide-pulsing experiment (31). Furthermore, HLA-Cs do exist as free chains (without β2m and/or peptide) on the cell surface (32, 33). These findings further strengthen our hypothesis that the unfolding at the α2 region may have released the peptide, thereby causing the HLA to be unstable, rendering it unsuitable for any downstream cytotoxic mechanisms when bound by the non-pathogenic Abs. The stability of HLA Class I peptide is crucial for immunodominance in protection against certain diseases such as HIV, particularly for the recognition of T cells (34). If our hypothesis is true, then T cell-mediated rejection would also be avoided, giving this antibody a protective role instead.

Another distinct difference that allowed us to distinguish the binding of pathogenic versus non-pathogenic antibodies was in terms of the overall structural stability of the HLA–Ab complex. The binding of pathogenic Abs resulted in a stable HLA–Ab complex, which can be observed across the HLA molecule throughout the four time points but not when bound by the non-pathogenic Abs.

This finding suggests that the structural stability of the HLA–Ab complex could be crucial in determining pathogenicity. A few studies have reported that the binding of neutralizing Abs to pathogens was found to stabilize the complex structure. Zhu et al. reported that the binding of a neutralizing Ab was found to stabilize the diphtheria toxin conformation when analyzed on HDX–MS (23). Another study reported the binding of an anti-hemagglutinin Fab reduced the conformational flexibility of the antigen, which led to the mechanism that is believed to be involved in the neutralization (35). These studies further highlight the possible correlation of the structural stability of the antigen–Ab complex with pathogenicity.

This study has several limitations. First, the HLA monomers were used for binding assay on ELISA and the analysis of binding kinetics. This may not represent the actual binding of the antibodies to HLAs presented on the cell surface. The second limitation is that we used a single isotype, IgG1, to study, while IgG3 is also known to mediate both CDC and ADCC. Our HDX data only studied four of the eight antibodies, representing two from each group, which is a limitation considering the small sample number. However, the results were derived from three independent experiments, with any difference observed in the deuterium uptake considered significant. Identifying binding epitopes will provide us with a better understanding of the correlation of antibody binding and its correlation with pathogenicity. We made a few attempts with crystallography but did not yield any results, as HLA-C, especially HLA-C*07, is known to be less stable compared to its counterparts HLA-A and HLA-B alleles (33).

One of the biggest weaknesses of the current clinical platform (Luminex) that is used for measuring DSAs is that it is only able to identify the presence of DSAs, but it does not inform if the patient is at risk of rejection because of our inability to discriminate the pathogenic antibody. We believe that this study is the first step to understanding the biological determinants of pathogenic antibodies, which could potentially be translated into a clinical platform that will allow us to stratify high-risk individuals. Some of the antibodies we characterized in this project have therapeutic potential. If our hypothesis on the unfolding causing the peptide to fall off is accurate, then the non-pathogenic antibodies could potentially be used to protect the graft. In addition to not being able to cause CDC or ADCC, the instability that was caused by these antibodies could also render the HLA unsuitable for other cell-mediated rejection. In addition, if any of the antibodies that we isolated have a binding epitope on the antigen-binding cleft, then it could potentially be utilized to block T cells from binding, thereby inhibiting T cell-mediated rejection.

In summary, our results reiterate that a mere binding of antibodies to HLA on the cell surface does not induce Fc-mediated immune responses as observed in the activity of the non-pathogenic Abs. In addition, antibodies with higher affinities could potentially be harmless. Our results indicated that pathogenicity may not be contributed by a single characteristic. We, however, found that antigen–antibody complex density seems to be important in influencing pathogenicity. All pathogenic antibodies reported high antigen–antibody complex density in comparison to the non-pathogenic antibodies. The fact that Ab 2E3, which had similar binding kinetics as the non-pathogenic antibodies such as 2G11 and 3E2, were able to induce cytotoxicity highlights the importance of antigen–antibody complex density in determining pathogenicity. This revelation is unsurprising since high antigen–antibody complex density can promote hexamer formation where the Fc region of the Abs is bonded non-covalently. Hence, regardless of the kinetics, even with fast off-rates, the Abs are held together through this formation. Furthermore, HDX–MS, which was performed to investigate the biophysical properties of the HLA–antibody interaction, revealed the enhanced structural stability of the HLA–Ab complex when bound by the pathogenic Abs. Cumulatively, our investigation into the characteristics of pathogenic antibodies revealed that factors that promote the ability of complex formation between the HLA–antibody and the structural stability of that complex directly influence pathogenicity.

## Data Availability

The original contributions presented in the study are included in the article/**Supplementary Material**. Further inquiries can be directed to the corresponding author.
