# Supplementary material for: Antigen–antibody complex density and antibody-induced HLA protein unfolding influence Fc-mediated antibody effector function
Source: Front Immunol. 2024 Dec 18;15:1438285. doi: 10.3389/fimmu.2024.1438285 (PMC11688311; doi:10.3389/fimmu.2024.1438285)
Supplement: Supplementary file 1 [file DataSheet1.docx]

**Supplementary figures:**

**Figure 1:**

**A**

**1A6-IgG1**

**1H2-IgG1**

**2E3-IgG1**

**4B5-IgG1**

**1B2-IgG1**

**2H7-IgG1**

**2G11-IgG1**

**3E2-IgG1**

**M**

**kDa**

**50**

**25**

**B**

**Figure 1:** Antibody discovery**. A)** Results from polyclonal ELISA from panning against HLA-A*11:01, -B*40:01 and -C*07:02. **B)** SDS-PAGE image of purified alloantibodies.

**Table 1: Kinetic measurements for all the 8 alloantibodies.** KD of IgGs are based on 1:1 Langmuir fitting. KD, k_on_ and k_off_ values are the mean and standard deviation of two independent experiments.

|  | **Antigen (HLA)** | **IgG1** | **K_D_ (M** x 10^-8^**)** | **k_on_ (1/Ms x** 10^4^**)** | **k_off_ (1/s x** 10^-4^**)** |
| --- | --- | --- | --- | --- | --- |
| **Pathogenic** | C*07:02 | 1A6 | 64.3 ± 37 | 13.1 ± 8.2 | 686 ± 39 |
|  | C*07:02 | 1H2 | 35.7 ± 12 | 24.9 ± 1.8 | 885 ± 35 |
|  | A*11:02 | 2E3 | 8.07 ± 0.81 | 0.45 ± 0.20 | 3.70 ± 0.21 |
|  | B*40:01-Streptavidin complex | 4B5 | 2.08 ± 0.26 | 17.5 ± 6.2 | 35.5 ± 8.4 |
| **Non-Pathogenic** | C*07:02 | 1B2 | 2.49 ± 0.71 | 1.53 ± 0.09 | 3.76 ± 0.86 |
|  | C*07:02 | 2H7 | 1.76 ± 0.13 | 2.16 ± 1.17 | 3.69 ± 1.75 |
|  | C*07:02 | 2G11 | 3.94 ± 0.29 | 0.93 ± 0.02 | 3.66 ± 0.16 |
|  | C*07:02 | 3E2 | 3.37 ± 0.44 | 0.96 ± 0.08 | 3.24 ± 0.13 |

**Figure 2**

**A**

**B**


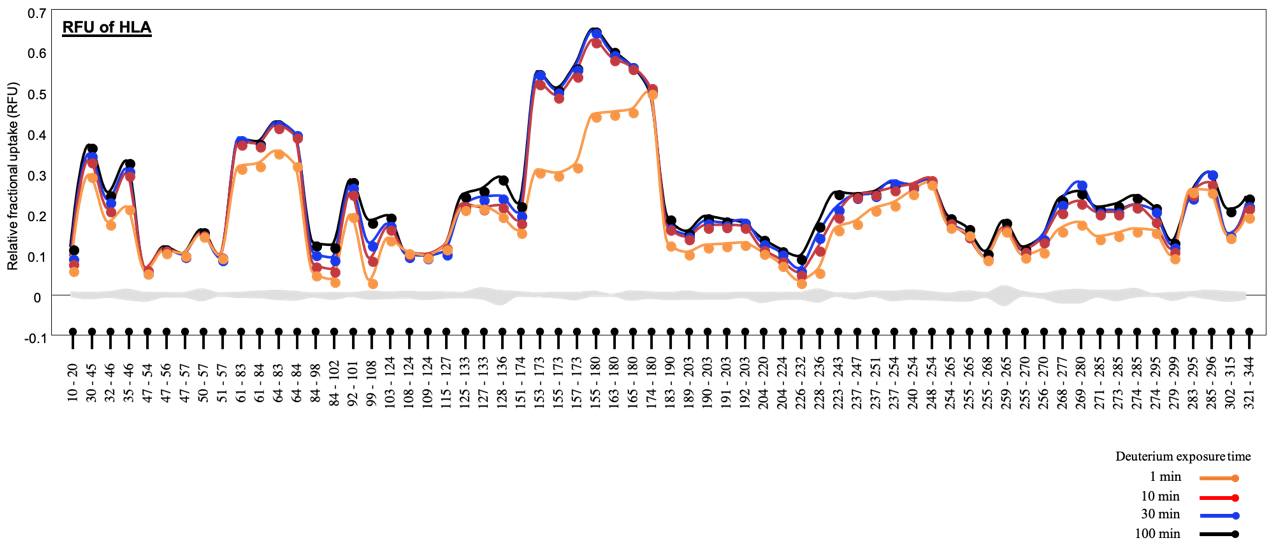


Figure 2: HDX profile of ‘apo’ HLA-C*07:02. (**A)** Relative fractional uptake (RFU) of HLA. **(B)** Sequence coverage of HLA; with 64 peptides identified that covered 82% of the sequence.
